# Opposite Incidence Trends for Differentiated and Medullary Thyroid Cancer in Young Dutch Patients over a 30-Year Time Span

**DOI:** 10.3390/cancers13205104

**Published:** 2021-10-12

**Authors:** Chantal A. Lebbink, Medard F. M. van den Broek, Annemiek B. G. Kwast, Joep P. M. Derikx, Miranda P. Dierselhuis, Schelto Kruijff, Thera P. Links, A. S. Paul van Trotsenburg, Gerlof D. Valk, Menno R. Vriens, Annemarie A. Verrijn Stuart, Hanneke M. van Santen, Henrike E. Karim-Kos

**Affiliations:** 1 Department of Pediatric Endocrinology, Wilhelmina Children’s Hospital, University Medical Center Utrecht, 3508 AB Utrecht, The Netherlands; C.A.Lebbink@umcutrecht.nl (C.A.L.); A.A.Verrijnstuart@umcutrecht.nl (A.A.V.S.); H.M.vanSanten@umcutrecht.nl (H.M.v.S.); 2 Princess Máxima Center for Pediatric Oncology, 3508 AB Utrecht, The Netherlands; M.P.Dierselhuis@prinsesmaximacentrum.nl; 3 Department of Endocrine Oncology, University Medical Center Utrecht, 3508 GA Utrecht, The Netherlands; M.F.M.vandenBroek-10@umcutrecht.nl (M.F.M.v.d.B.); G.D.Valk@umcutrecht.nl (G.D.V.); 4 Department of Research and Development, Netherlands Comprehensive Cancer Organisation (IKNL), 3511 DT Utrecht, The Netherlands; a_kwast@hotmail.com; 5 Department of Pediatric Surgery, Emma Children’s Hospital, Amsterdam University Medical Centers, University of Amsterdam, 1105 AZ Amsterdam, The Netherlands; j.derikx@amsterdamumc.nl; 6 Department of Surgery, University Medical Center Groningen, University of Groningen, 9713 GZ Groningen, The Netherlands; s.kruijff@umcg.nl; 7 Department of Endocrinology, University Medical Center Groningen, University of Groningen, 9713 GZ Groningen, The Netherlands; t.p.links@umcg.nl; 8 Department of Pediatric Endocrinology, Emma Children’s Hospital, Amsterdam University Medical Centers, University of Amsterdam, 1105 AZ Amsterdam, The Netherlands; a.s.vantrotsenburg@amsterdamumc.nl; 9 Department of Endocrine Surgical Oncology, University Medical Center Utrecht, 3508 GA Utrecht, The Netherlands; mvriens@umcutrecht.nl

**Keywords:** thyroid cancer, epidemiology, incidence, survival, children, adolescents, young adults

## Abstract

**Simple Summary:**

Thyroid cancer is a rare disease in childhood; however, its incidence is rising. Thyroid cancer consists of three main types: Papillary thyroid cancer (PTC), follicular thyroid cancer (FTC), and medullary thyroid cancer (MTC). The aim of our retrospective study was to investigate the incidence and survival trends of these three thyroid cancer types in Dutch children, adolescents, and young adults over a 30-year life span. In total, 839 patients aged 0–24 years had been diagnosed with thyroid cancer between 1990 and 2019. The incidence of PTC increased significantly over time, the incidence of FTC showed a stable trend, while the incidence of MTC decreased significantly. Overall, the 10-year survival rates over the last decades were high (>95%) for PTC, FTC, and MTC in young individuals.

**Abstract:**

Thyroid cancer is the most common endocrine malignancy in children. A rising incidence has been reported worldwide. Possible explanations include the increased use of enhanced imaging (leading to incidentalomas) and an increased prevalence of risk factors. We aimed to evaluate the incidence and survival trends of thyroid cancer in Dutch children, adolescents, and young adults (0–24 years) between 1990 and 2019. The age-standardized incidence rates of differentiated thyroid cancer (DTC, including papillary and follicular thyroid cancer (PTC and FTC, respectively)) and medullary thyroid cancer (MTC), the average annual percentage changes (AAPC) in incidence rates, and 10-year overall survival (OS) were calculated based on data obtained from the nationwide cancer registry (Netherlands Cancer Registry). A total of 839 patients aged 0–24 years had been diagnosed with thyroid carcinoma (PTC: 594 (71%), FTC: 128 (15%), MTC: 114 (14%)) between 1990 and 2019. The incidence of PTC increased significantly over time (AAPC +3.6%; 95%CI +2.3 to +4.8), the incidence rate of FTC showed a stable trend ((AAPC −1.1%; 95%CI −3.4 to +1.1), while the incidence of MTC decreased significantly (AAPC: −4.4% (95%CI −7.3 to −1.5). The 10-year OS was 99.5% (1990–1999) and 98.6% (2000–2009) in patients with DTC and 92.4% (1990–1999) and 96.0% (2000–2009) in patients with MTC. In this nationwide study, a rising incidence of PTC and decreasing incidence of MTC were observed. For both groups, in spite of the high proportion of patients with lymph node involvement at diagnosis for DTC and the limited treatment options for MTC, 10-year OS was high.

## 1. Introduction

Thyroid cancer is rare during early childhood (<10 years of age) [1,2]; however, it is the eighth most frequently diagnosed cancer among adolescents (15–19 years) [3]. Thyroid cancer accounts for 2–6% of all pediatric malignancies, making it the most common endocrine cancer in children [4,5]. In addition, it is the second most common cancer in adolescent girls, due to a strong female predominance of differentiated thyroid carcinoma (DTC) which usually manifests during puberty [3,6]. Thyroid cancer comprises a wide spectrum of histological subtypes; on the one hand, there are two types of DTC originating from follicular cells (papillary and follicular thyroid cancer (PTC and FTC, respectively)), while on the other hand, medullary thyroid carcinoma (MTC) derives from parafollicular C-cells. The papillary subtype represents the large majority of cases with pediatric thyroid cancer (83%), followed by FTC (10%) and MTC (5%) [1,7]. In pediatric patients with DTC, lymph node involvement and distant metastases at the time of diagnosis occur more frequently than in adults [2]. Nevertheless, pediatric DTC has an excellent prognosis [8]. Pediatric MTC is not susceptible to radio-iodine treatment, and is associated with worse survival [7,9]. For this reason, in familiar cases such as the multiple endocrine neoplasia type 2 (MEN2) syndrome, prophylactic thyroidectomy is advised [10].

A rising incidence in pediatric thyroid cancer—especially PTC—over the last decades has been reported in several studies and matches epidemiological findings of thyroid malignancy in adults [8,11]. In the USA, the incidence of thyroid cancer in patients aged 0–19 years showed a gradual annual percent change (APC) of +1.1% during 1973–2006, while it markedly increased thereafter (APC 2006–2013: +9.6%) [8]. Studies in Europe and South Korea have demonstrated comparable results [12,13]. There is an ongoing debate about the underlying mechanisms that may explain this phenomenon. Some suggest it is attributable to overdiagnosis, driven by the combination of the expanding usage of imaging studies, enhanced imaging techniques, in combination with the high prevalence of indolent differentiated thyroid tumors even in the juvenile population [14], while others argue that the concurrently increased incidence of large tumors and advanced-stage disease is proof of a ‘true’ rise in pediatric DTC [8,15]. Suggested explanations for a true rise in children are the increased obesity prevalence and radiation exposure as a consequence of environmental radiation or after treatment for childhood cancer [16,17,18].

The Netherlands has a comprehensive national cancer registry (Netherlands Cancer Registry, NCR), which creates the opportunity to investigate the true incidence trends of thyroid malignancy with great accuracy. We aimed to retrospectively evaluate pediatric thyroid cancer incidence and survival trends from 1990 to 2019, based on patient and tumor characteristics among patients aged 0–17 years in the Netherlands using the population-based data of the NCR. Young adults (18–24 years) were also included as a (post-pubertal) comparative group, embodying the youngest patients treated in adult oncology centers.

## 2. Materials and Methods

### 2.1. Study Population

All Dutch patients below 25 years of age diagnosed with a malignant thyroid carcinoma from January 1990 to December 2019 were selected retrospectively from the Netherlands Cancer Registry (NCR).

### 2.2. Definitions

Thyroid carcinoma cases were classified according to the International Classification of Diseases for Oncology, Third Edition (ICD-0-3) by topography (C73) and histology: Papillary (ICD-O-3 M8050, M8140, M8201, M8260, M8340-44, M8350, and M8504), follicular (ICD-O-3 M8290, M8330-32, M8335, and M8339), medullary (ICD-O-M8345, M8510-11) thyroid carcinoma, and others (ICD-O-3 M8000, M8337, and M8346) [19]. Only thyroid carcinomas with malignant behavior (i.e., 5th digit of the morphology code/3) were included. No cases were diagnosed with an anaplastic thyroid carcinoma during our study period. Tumor staging was recorded according to the TNM (Tumor, Node, Metastasis) classification system of the Union for International Cancer Control (UICC) [20]. The edition applicable at the time of diagnosis of thyroid carcinoma was used. In the case of a missing pathological TNM classification, the clinical TNM was used.

Patients were classified as treated in a university hospital if they received thyroidectomy and/or radio-iodine treatment in a university hospital. 

### 2.3. The Netherlands Cancer Registry

The nationwide population-based NCR is maintained and hosted by the Netherlands Comprehensive Cancer Organisation (IKNL) and has had national coverage since 1989 with a completeness value of at least 96% of all newly diagnosed malignancies in the Netherlands [21]. The NCR relies on comprehensive case notification through the Nationwide Network and Registry of Histopathology and Cytopathology, and the National Registry of Hospital Discharges. Retrospectively, data are extracted on patient, tumor, and treatment characteristics. Information on vital status (i.e., alive, dead, or emigration) was obtained by annual linkage of the NCR with the Personal Records Database (BRP) that holds vital statistics on all residents in the Netherlands. The last linkage was on 1st February 2021.

### 2.4. Statistical Analyses

Characteristics of the study population were described as percentages in relation to three periods of diagnosis: 1990–1999, 2000–2009, and 2010–2019. In addition, patient characteristics were analyzed for the following age groups: 0–17 (children and adolescents) and 18–24 years (young adults). Patients diagnosed with DTC and PTC were further divided into 0–9 (children), 10–14 (children), 15–17 (adolescents), and 18–24 years. This was not possible for FTC and MTC due to the low number of cases. Differences among categorical variables were tested with the χ^2^ tests or the Monte Carlo estimate for the Exact test in case of small numbers.

Annual incidence rates were calculated per million person-years, using the annual mid-year population size as obtained from Statistics Netherlands. Rates were age-standardized according to the age structure of the World standard population for the age ranges 0–9, 0–17, and 0–24 years [22]. Incidence rates were presented in the figures as three-year moving averages by taking the average of the rates of each given year and the rates on either side of it. Changes in incidence over time were evaluated by calculating the average annual percentage change (AAPC) along with the corresponding 95% confidence intervals (CI). AAPC was derived from linear regression modelling, using the calendar year as a continuous variable [22]. The Joinpoint regression program (version 4.5.0.1; https://surveillance.cancer.gov/joinpoint/, accessed on 24 June 2021) was used to check for trend transitions during the study period [23]. The null hypothesis assumed that the AAPC was constant throughout the study period. The permutation test was used to determine the number of joinpoints by default set to a maximum of four [24]. 

Survival time was calculated as the time elapsed between the date of diagnosis and the date of death due to any cause (event) or censoring (i.e., loss to follow-up, emigration, or 1 February 2021), whichever came first. Traditional actuarial survival analysis was used to calculate overall survival (OS) at 10 years after diagnosis. OS was used instead of relative OS, which is an estimation of the disease-specific survival, because competing causes of death are rare among young cancer patients in developed countries such as the Netherlands [25]. Kaplan–Meier curves and the logrank test were used for visualization and comparison of survival between DTC and MTC, respectively. Additional survival analysis to evaluate changes in survival over time was not possible due to the low number of events.

Incidence analyses were performed using SAS software (SAS system 9.4, SAS Institute, Cary, NC, USA), whereas STATA/SE 16.1 (StataCorp LP, College Station, TX, USA) was used for survival analyses. A *p*-value < 0.05 was considered statistically significant.

## 3. Results

**Figure 1:**
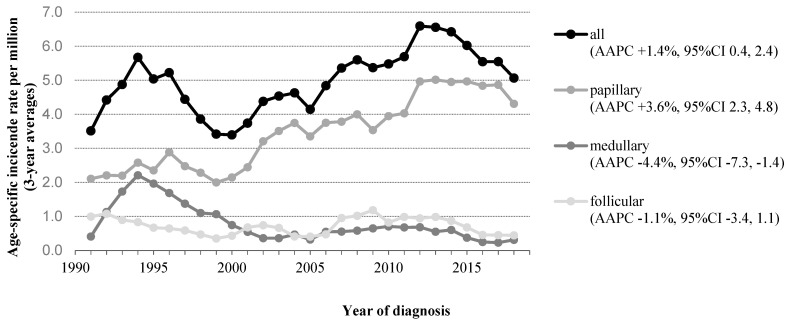
Time trends in incidence of patients aged 0–24 years with thyroid carcinoma in The Netherlands, 1990–2019. Abbreviations: AAPC, average annual percent change; CI, confidence interval. Three-year moving averages of the age-standardized incidence rate of thyroid carcinoma (standardized according to the World Standard Population) are shown. AAPC was estimated from a regression line, which was fitted to the natural logarithm of the rates using the year of diagnosis as a regressor variable.

A total of 839 children, adolescents, and young adults aged 0–24 years (Table S1) had been diagnosed with thyroid carcinoma between 1990 and 2019. The most common histopathological tumor subtype was PTC, accounting for 71% of the cases (*n* = 594). FTC and MTC were found in 15% (*n* = 128) and 14% (*n* = 114) of the cases, respectively. Three patients had been diagnosed with a mixed/other histologic tumor type. Overall, the incidence of thyroid carcinoma increased in children/adolescents/young adults between 1990 and 2019 with an AAPC of +1.4% (95%CI 0.4 to 2.4) (Figure 1). Further results are described by subgroup: DTC (consisting of PTC and FTC) and MTC.

### 3.1. Differentiated Thyroid Carcinoma (DTC)

**Figure 2:**
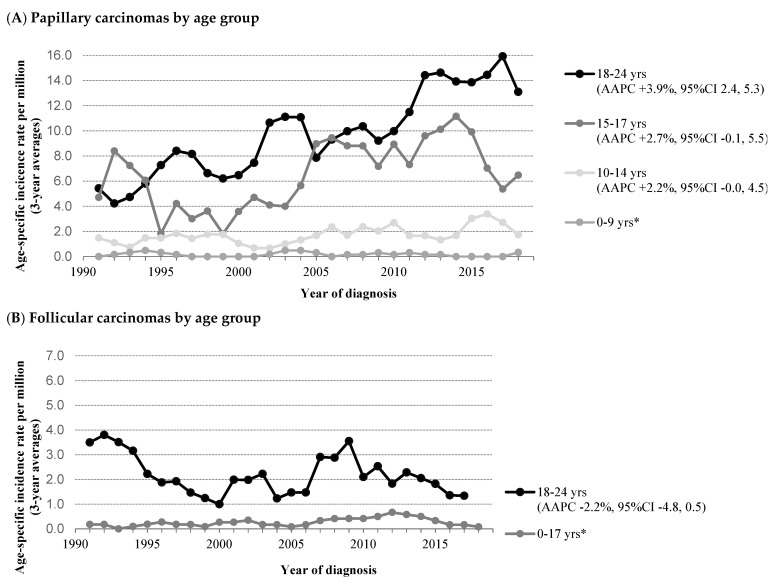

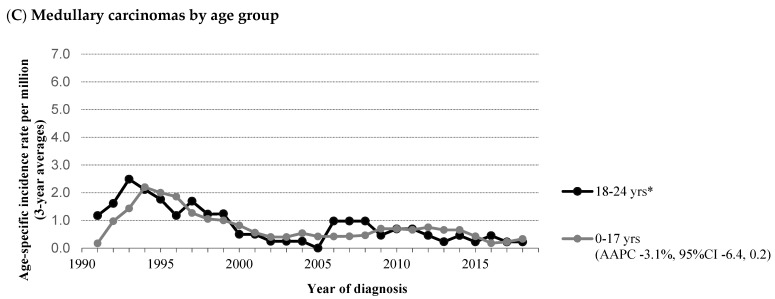
Time trends in incidence of patients aged 0–24 years with thyroid carcinoma by histology and age in the Netherlands, 1990–2019. (**A**) Papillary thyroid carcinoma. (**B**) Follicular thyroid carcinoma. (**C**) Medullary thyroid carcinoma. Abbreviations: AAPC, average annual percent change; CI, confidence interval. Three-year moving averages of the age-specific incidence rate of thyroid carcinoma are shown. The incidence rates of the patients 0–9 and 0–17 years are age-standardized according to the World Standard Population. AAPC was estimated from a regression line, which was fitted to the natural logarithm of the rates using year of diagnosis as a regressor variable. * Estimation of a reliable average annual percentage change was not possible because of *n* = 0 in >5 incidence years.

In a 30-year time span, 722 children, adolescents, and young adults had been diagnosed with DTC. The incidence of DTC in children/adolescents/young adults increased significantly over time, from 3.1 per million person-years (1990–1999) to 5.3 per million person-years (2010–2019), with an AAPC of +2.6% (95%CI +1.6 to +3.7) (Figure 1). No join points were identified, which implicates a steady increase in incidence over time. Similar shifts in incidence were found, when specifically looking into PTC; the incidence of PTC increased significantly over time (AAPC +3.6%; 95%CI +2.3 to +4.8). In contrast, the incidence rates among FTC showed a stable trend, although the number of patients diagnosed with FTC was very low. The age-specific incidence rates are presented in Figure 2A,B. The incidence of DTC among boys as well as girls increased significantly over time (Table S2). When focusing on age subgroups, the increasing incidence of PTC was seen in all age groups ≥10 years; however, this was only significant in young adults (*p* < 0.001).

**Figure 3:**
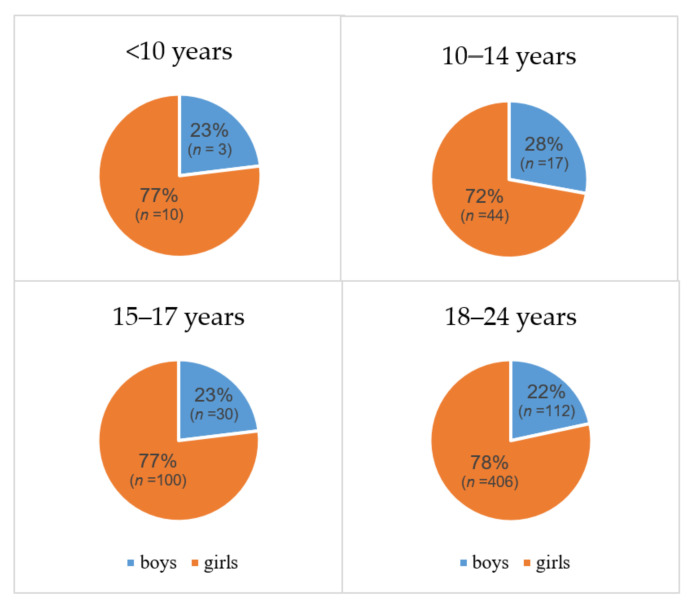
Sex distribution of differentiated thyroid carcinoma within different age groups in the Netherlands, 1990–2019. Sex distribution of differentiated thyroid carcinoma of the age groups <10, 10–14 years, 15–17 years, and 18–24 years. Both percentage and the absolute number of patients are shown.

Of all patients with DTC, 28% (*n* = 204) were <18 years of age (Table 1A); only 2% (*n* = 13) of the cohort was aged <10 years at diagnosis of DTC. The age distribution of DTC did not differ over time. Girls were more often affected than boys (78% vs. 22%, respectively) regardless of age (Figure 3). DTC as second primary cancer was observed in 2% (*n* = 18, all PTC) of the patients. Most patients with DTC (42%) were found to have a T2 stage tumor. The distribution of T-stage changed significantly over time (*p* < 0.001), with a shift from T4 stage to T3 stage and from T2 stage to T1 stage. In more than 40% (*n* = 300) of the patients with DTC, lymph node metastases were found in the pathological report. Children and adolescents were diagnosed with lymph node metastases more often compared to young adults (54% vs. 40%, *p* = 0.001). Significantly more lymph node metastases were reported in the period 2000–2009 (52%, *p* < 0.02). Distant metastases were reported in 3% of the patients with DTC in total, and were more frequently found in children (15% vs. 2% in older patients, *p* < 0.001). The characteristics of DTC by age group are presented in Table S3.

Over the years, patients with DTC were treated at a university hospital significantly more often (48% in 1990–1999, 67% in 2000–2009, and 73% in 2010–2019, *p* < 0.001). This shift was especially noticeable in children and adolescents (0–17 years) (Figure S1).

The median follow-up of all patients with DTC was 12.2 years. A total of 14 patients with DTC died during follow-up (12 PTC, 2 FTC, 5 of them within 10 years of follow-up), from which the cause of death was unknown. The 10-year overall survival was comparable between 1990–1999 and 2000–2009 (99.5% vs. 98.6%, respectively).

### 3.2. Medullary Thyroid Carcinoma (MTC)

A total of 114 patients had been diagnosed with MTC during the study period. The incidence of MTC significantly decreased from 1.3 per million person-years in 1990–1999 to 0.5 per million person-years in 2010–2019 (AAPC: −4.4% (95%CI −7.3 to −1.5)) (Figure 1). The incidence showed a downward near-significant trend for the younger age group (<18 years) (AAPC: −3.1% (95%CI −6.4 to +0.2), whereas estimation of a reliable AAPC was not possible for the young adults due to the low incidence in this group (Figure 2C). The age at diagnosis and sex distribution did not change significantly over time. Tumor size distribution remained stable during 1990–2019, while the proportion of MTC with regional lymph node involvement showed a significant increase over time (*p* = 0.045) (Table 1B). As shown in Figure 1 and Figure 2C, the incidence rates of MTC showed a peak around 1994. Joinpoint analyses could not be performed due to the small number of events.

Boys and girls were equally affected (48% vs. 52%, respectively). In contrast to DTC, the majority of cases had been identified in childhood and adolescence (68% at age 0–17 years). Young adults diagnosed with MTC suffered from more advanced disease upon diagnosis than younger patients, illustrated by the significantly higher T-stage (*p* = 0.01) and the higher proportion of patients with lymph node involvement (54% in young adults vs. 17% in patients <18 years, *p* < 0.001, Table S2B). Likewise, young adults seemed to be diagnosed with a metastatic disease more often than children/adolescents (13% vs. 4% respectively), but this trend did not reach statistical significance (*p* = 0.14).

Over the years, patients with MTC were treated at a university hospital significantly more often (68% in 1990–1999, 92% in 2000–2009, and 96% in 2010–2019, *p* = 0.003). This shift in MTC care was detected in both children/adolescents and young adults (Figure S1).

**Figure 4:**
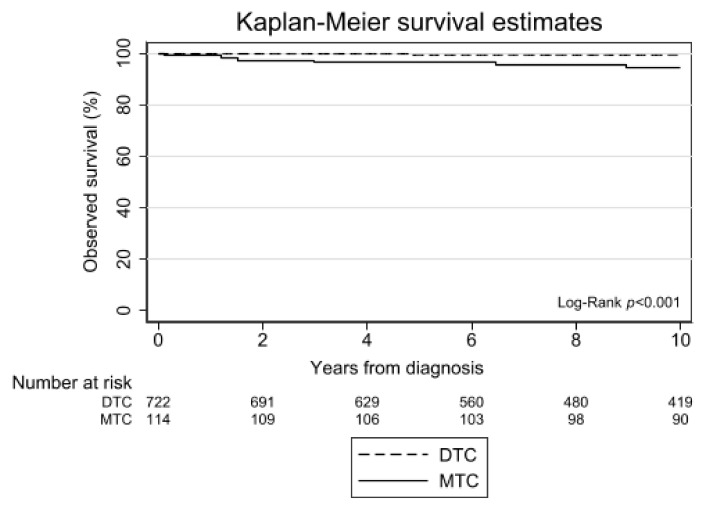
Observed survival of patients, aged 0–24 years with thyroid carcinoma in the Netherlands, 1990–2019. Abbreviations: DTC, differentiated thyroid carcinoma; MTC, medullary thyroid carcinoma. Survival time was calculated as the time elapsed between the date of diagnosis and the date of death due to any cause (event) or censoring (i.e., loss to follow-up, emigration, or 1 February 2021), whichever came first. The log rank test showed a significantly different 10-year survival between DTC and MTC: *p* < 0.001.

The median follow-up of all patients with MTC was 21.1 years. A total of 12 patients with MTC died during follow-up (six within 10 years after diagnosis), from which the cause of death was unknown. The 10-year overall survival was 92.4% for patients diagnosed in the period 1990–1999 and 96.0% for patients diagnosed in 2000–2009. Patients (all ages) with MTC experienced significantly worse survival than DTC (*p* < 0.001) (Figure 4).

## 4. Discussion

This nationwide study, spanning three decades, shows opposite incidence trends for DTC and MTC in young individuals: Increasing incidence of DTC and decreasing incidence of MTC. In addition, the very good prognosis for both DTC as well as MTC in young patients (0–24 years) is confirmed, despite the frequent presence of advanced disease.

### 4.1. Differentiated Thyroid Carcinoma (DTC)

The incidence rate of DTC in our cohort was comparable to previous studies [7,8,11,13,26], and the incidence of DTC seemed to increase in all age groups, with DTC being most frequently diagnosed in patients >18 years [13]. This is mainly attributed to the increase in PTC over time. In accordance with two previous studies, stable incidence numbers of FTC over time were seen [8,13]. 

A preponderance of girls has been a persistent finding in previous studies reporting on pediatric DTC [11]. It is suggested that this difference may be induced by estrogen as estrogen is a potent growth factor for both benign and malignant thyroid cells [27]. With this in mind, the fact that a predominance of affected girls was also found in pre-pubertal girls <10 years was surprising. Of these girls, six were <8 years and seven were aged 8–10. Possibly, the girls aged >8 years already had some activation of the gonadal axis contributing to this increased female/male ratio; unfortunately, data on the pubertal stage of the children could not be retrieved. The fact, however, that in the age group of 4–7 years the girls were also overrepresented may indeed confirm predominance also in pre-pubertal girls. This finding does not stand on its own [28,29]. Lazar et al. described a male/female ratio of 3/7 in pre-pubertal children. Farahati et al. also described a male/female ratio of 1/7 in children <8 years [28]. For the predominance of pubertal and pubertal girls, several hypotheses may be suggested: In these young girls, estrogen exposure during mini-puberty may have been a trigger for the development of DTC at such young age. In addition, it may be considered that estrogen derived from adipose tissue may have contributed; for this reason, in future studies, BMI should be taken into account. Of course, the small patient numbers make it impossible to draw conclusions and this must be further investigated in larger cohorts.

We found lymph node metastases in more than 40% of the patients with DTC, comparable to previous reports (51%) [26]. However, relatively few patients with distant metastasis were found (3% versus 7.8–7.9% [1,26]). Possibly, this might be explained by a delay in diagnosis as a result of an overall poorer insurance status compared to our cohort [30]. In line with previous studies, children and adolescents in our cohort were significantly more often diagnosed with more advanced disease, compared to young adults [2].

Over time, the T-stage of DTC patients at diagnosis shifted from T4 to T3 and from T2 to T1, suggesting that patients were diagnosed at an earlier stage. This shift in stage at diagnosis may be the result of improved quality and increased use of diagnostic imaging tools. Furthermore, the transition in the TNM classification system editions over time may have influenced our results, since we based the TNM stage on the TNM edition applicable during the year of diagnosis. For example, the altered definition of T2 (tumor size >1 cm to ≤4 cm during 1990–2002 vs. >2 cm to ≤4 cm afterwards) could presumably explain the shift from T2 towards T1 tumors in recent years.

### 4.2. Medullary Thyroid Carcinoma (MTC)

Contrary to DTC, the incidence of MTC decreased significantly during the study period. The literature on incidence trends of pediatric MTC is very limited. In 2018, Schmidt Jensen et al. described 27 patients aged 0–24 years with MTC and found no significant change in incidence over time (1980–2014) [13]. A year later, Qian et al. reported an unchanged rate of MTC throughout the study period (1973–2013) in a cohort aged 0–19 years with MTC (cohort size unknown) [8]. A possible explanation for the difference in incidence trends found in the Danish, American, and now Dutch populations are the small number of patients in the Danish and American cohorts. Another explanation for this could be a difference in approach to timely genetic counseling and, subsequently, preventive thyroidectomies at a young age in children diagnosed with MEN2. Also in contrast to DTC—but in line with previous research—MTC was diagnosed most frequently in patients <18 [7]. Patients >18 years more often presented with advanced disease, which may reflect late diagnosis in non-familiar or not-yet-recognized familiar cases. Genetic syndromes harboring an increased risk for MTC may be difficult to recognize, contributing to the delay in diagnosis of MTC [31].

The incidence of MTC peaked around 1994 and dropped afterwards. Synchronously, patients with MTC were found to have more advanced disease upon diagnosis in recent years. These findings can possibly be explained by the introduction of pre-symptomatic DNA screening in children from MEN2A families and prophylactic thyroidectomy in children with a high risk of MTC, which became common practice after the identification of germline mutations in the *RET* gene as the origin of MEN2 syndromes in the early 1990s [32,33]. In the first years after the implementation of *RET* mutation screening, many children from MEN2A families were identified with local MTC, which resulted in the earlier mentioned peak incidence around 1994. After this first “wave”, DNA screening in early childhood prompted an early prophylactic thyroidectomy before the onset of MTC in the majority of children with MEN2A, explaining the declined incidence of MTC in the following years. On the contrary, children with MEN2B are unfortunately often not diagnosed until after the development of symptomatic (advanced) MTC, because *RET* mutations occur as *de novo* in 75–90% of MEN2B patients [10,34]. Therefore, the implementation of DNA screening presumably did not affect the incidence of MEN2B-related MTC on a large scale. Together with the decrease in MEN2A-related MTC, this may have led to an increased proportion of (late-recognized) MTC in the context of MEN2B. This may also explain our finding of the higher percentage of MTC patients with lymph node involvement found in more recent years. In addition, MTC within the context of MEN2B is known to occur even earlier in life and with more aggressive behavior when compared to MEN2A. 

### 4.3. Site of Treatment

Over the years, patients with thyroid carcinoma—both DTC and MTC—were more often treated in a university hospital, reflecting centralization of healthcare. Centralization of care is an important step in improving care for children, adolescents, and young adults with rare diseases such as thyroid carcinoma in order to optimize diagnostics, management, and outcomes while minimizing the long-term adverse consequences [35]. We could not detect an improvement in 10-year OS over the years. Future studies will have to evaluate whether the number of adverse effects of treatment, such as hypoparathyroidism, have decreased with increasing centralization.

### 4.4. Strengths and Limitations

The major strengths of this study include its national coverage and standardized data collection. These elements resulted in the availability of generalizable and reliable data with a low risk of (information) bias. Furthermore, the long follow-up period allowed us to analyze trends in incidence and outcome over a longer period of time, including the effect of the implementation of *RET* mutation analysis in MEN2A families.

Our study has several limitations. First, the exact causes of death were unknown. The causes-of-death database hosted by Statistics Netherlands (CBS) is not linked with the NCR on a routine basis. Therefore, we have checked the number of deceased patients in our study with the number of Dutch inhabitants who died from thyroid carcinoma at a young age derived from the independent causes-of-death statistics (source: Statistic Netherlands, CBS), and these numbers did not differ. Moreover, the absence of causes of death will have a limited effect on our survival outcomes as competing causes of death are rare among young cancer patients in developed countries such as the Netherlands [25]. Second, the NCR used clinical (including ultrasound, computed tomography imaging, and functional imaging if available) and pathological data for stage registration until total thyroidectomy, which may have resulted in incorrect data about lymph node status or the presence of distant metastases found at ^131^I- scanning post-surgery. This may have led to an underestimation of the ‘true’ number of patients with positive lymph nodes or distant metastases. Furthermore, for DTC specifically, changes in the tumor staging system over time may have influenced the results. For MTC specifically, the low incidence and mortality numbers prevented us from performing further analyses into factors possibly related to the incidence or survival of MTC. Finally, information about germline *RET* mutations in patients with MTC, the family history of patients, and the incidence (trends) of premalignant C-cell hyperplasia would have helped to further elucidate the results of this study, but these data were not available.

## 5. Conclusions

In summary, the presently reported outcomes of the national Dutch cohort demonstrate an increasing incidence of pediatric PTC between 1990 and 2019, with a shift towards smaller tumors. This may be a reflection of a true rise, or, alternatively, it may reflect the increased usage and quality of diagnostics such as ultrasound of the neck. In contrast, the incidence of MTC decreased during this period, presumably explained by the implementation of pre-symptomatic DNA analysis in MEN2A families in the early 1990s. Furthermore, despite a more advanced disease in children and adolescents compared to adults, the overall survival rates over the last decades remain high for both DTC and MTC in young individuals.

## Figures and Tables

**Table 1:** (**A**) Characteristics of differentiated thyroid carcinoma patients aged 0–24 years in the Netherlands, 1990–2019. (**B**) Characteristics of medullary thyroid carcinoma patients aged 0–24 years in the Netherlands, 1990–2019.

(A)										
Characteristics	Total		Average per Year	Period of Diagnosis						
				1990–1999		2000–2009		2010–2019		
	*N*	%	*N*	*N*	%	*N*	%	*N*	%	*p*-value
	722		24	186	26	223	31	313	43	
**Age**										0.75
0–9	13	2	0	4	2	5	2	4	1	
10–14	61	8	2	16	9	18	8	27	9	
15–17	130	18	4	27	15	46	21	57	18	
18–24	518	72	17	139	75	154	69	225	72	
Median age (in years, p25–p75)	20	(17–23)		21	(17–23)	20	(17–23)	20	(17–23)	0.23
**Sex**										0.98
boys	162	22	5	42	23	49	22	71	23	
girls	560	78	19	144	77	174	78	242	77	
**Histology**										0.002
papillary carcinoma	594	82	20	138	74	185	83	271	87	
follicular carcinoma	128	18	4	48	26	38	17	42	13	
**T stage ^a^**										<0.001
1	198	28	7	25	14	64	31	109	35	
2	292	42	10	101	57	82	39	109	35	
3	145	21	5	22	13	41	20	82	26	
4	62	9	2	28	16	22	11	12	4	
unknown (3% of total)	25		1	10		14		1		
**N stage ^a^**										0.02
0	379	56	13	98	59	99	48	182	59	
1	300	44	10	68	41	108	52	124	41	
unknown (6% of total)	43		1	20		16		7		
**Metastases ^a^**										0.41
no	606	97	20	140	97	161	95	305	97	
yes	20	3	1	4	3	8	5	8	3	
unknown (13% of total)	96		3	42		54		0		
**Thyroid carcinoma as second primary cancer**										0.65
yes	18	2	1	6	3	6	3	6	2	
no	704	98	23	180	97	217	97	307	98	
**(B)**										
**Characteristics**	**Total**		**Average per Year**	**Period of Diagnosis**						
				**1990–1999**		**2000–2009**		**2010–2019**		
	*N*	%	*N*	*N*	%	*N*	%	*N*	%	*p*-value
	114		4	66	58	25	22	23	20	
**Age**										0.67
0–17	78	68	3	43	65	18	72	17	74	
18–24	36	32	1	23	35	7	28	6	26	
Median age (in years, p25–p75)	13	(6–19)		13.5	(8–19)	11	(6–18)	11	(3–18)	0.32
**Sex**										0.18
boys	55	48	2	35	53	8	32	12	52	
girls	59	52	2	31	47	17	68	11	48	
**T stage ^a^**										0.35
1	83	78	3	48	79	19	83	16	70	
2	12	11	0	7	11	1	4	4	17	
3	6	6	0	2	3	1	4	3	13	
4	6	6	0	4	7	2	9	0	0	
unknown (6% of total)	7		0	5		2		0		
**N stage ^a^**										0.045
0	67	72	2	42	82	13	59	12	60	
1	26	28	1	9	18	9	41	8	40	
unknown (18% of total)	21		1	15		3		3		
**Metastases ^a^**										0.84
Yes	5	6	0	2	5	1	6	2	9	
no	75	94	3	37	95	17	94	21	91	
unknown (30% of total)	34		1	27		7		0		

Abbreviations: *N*, number. Characteristics of the study population were described as percentages in relation to the three periods of diagnosis: 1990–1999, 2000–2009, and 2010–2019. Differences among categorical variables were tested with the χ^2^ tests or the Monte Carlo estimate for the Exact test in case of small numbers. ^a^ Tumor staging was recorded according to the TNM (Tumor, Node, Metastasis) classification system of the Union for International Cancer Control (UICC). The edition applicable at the time of diagnosis of thyroid carcinoma was used.

## Data Availability

The dataset generated and analyzed for the current study is not publicly available due to the potential identifiable nature of the data. However, fully deidentified data can become available from the corresponding author on reasonable request.

## Supplementary Materials

The following are available online at https://www.mdpi.com/2072-6694/13/20/5104/s1, Figure S1. Proportion of patients with thyroid carcinoma aged <18 years and aged 18–24 years, treated at a university center, Table S1: Absolute number of cases thyroid cancer according to age in young Dutch patients over a 30-year time span, Table S2: Incidence of thyroid carcinoma in children, adolescents, and young adults aged 0–24 years in the Netherlands, 1990–2019, Table S3A: Characteristics of differentiated thyroid carcinoma patients aged 0–24 years in the Netherlands by age group, 1990–2019, Table S3B: Characteristics of medullary thyroid carcinoma patients aged 0–24 years in the Netherlands by age group, 1990–2019.
