# Correction: Lebbink et al. Opposite Incidence Trends for Differentiated and Medullary Thyroid Cancer in Young Dutch Patients over a 30-Year Time Span. *Cancers* 2021, *13*, 5104

**DOI:** 10.3390/cancers14122929

**Published:** 2022-06-14

**Authors:** Chantal A. Lebbink, Medard F. M. van den Broek, Annemiek B. G. Kwast, Joep P. M. Derikx, Miranda P. Dierselhuis, Schelto Kruijff, Thera P. Links, A. S. Paul van Trotsenburg, Gerlof D. Valk, Menno R. Vriens, Annemarie A. Verrijn Stuart, Hanneke M. van Santen, Henrike E. Karim-Kos

**Affiliations:** 1 Department of Pediatric Endocrinology, Wilhelmina Children’s Hospital, University Medical Center Utrecht, 3508 AB Utrecht, The Netherlands; c.a.Lebbink@umcutrecht.nl (C.A.L.); a.a.Verrijnstuart@umcutrecht.nl (A.A.V.S.); h.m.vanSanten@umcutrecht.nl (H.M.v.S.); 2 Princess Máxima Center for Pediatric Oncology, 3508 AB Utrecht, The Netherlands; m.p.dierselhuis@prinsesmaximacentrum.nl; 3 Department of Endocrine Oncology, University Medical Center Utrecht, 3508 GA Utrecht, The Netherlands; m.f.m.vandenBroek-10@umcutrecht.nl (M.F.M.v.d.B.); g.d.Valk@umcutrecht.nl (G.D.V.); 4 Department of Research and Development, Netherlands Comprehensive Cancer Organisation (IKNL), 3511 DT Utrecht, The Netherlands; a_kwast@hotmail.com; 5 Department of Pediatric Surgery, Emma Children’s Hospital, Amsterdam University Medical Centers, University of Amsterdam, 1105 AZ Amsterdam, The Netherlands; j.derikx@amsterdamumc.nl; 6 Department of Surgery, University Medical Center Groningen, University of Groningen, 9713 GZ Groningen, The Netherlands; s.kruijff@umcg.nl; 7 Department of Endocrinology, University Medical Center Groningen, University of Groningen, 9713 GZ Groningen, The Netherlands; t.p.links@umcg.nl; 8 Department of Pediatric Endocrinology, Emma Children’s Hospital, Amsterdam University Medical Centers, University of Amsterdam, 1105 AZ Amsterdam, The Netherlands; a.s.vantrotsenburg@amsterdamumc.nl; 9 Department of Endocrine Surgical Oncology, University Medical Center Utrecht, 3508 GA Utrecht, The Netherlands; mvriens@umcutrecht.nl

**Error in Figure/Table**

In the original article [1], there was a mistake in Figure 1 as published. In this figure, two years are missing (2000 and 2001). The AAPC values as previously published are correct. The corrected Figure 1 appears below.

In addition, in the original article, there was a mistake in Figure 2A–C as published. In these figures, two years are missing (2000 and 2001). The AAPC values were correct and do not require adjustment. The corrected Figure 2A–C appears below.

The authors apologize for any inconvenience caused and state that the scientific conclusions are unaffected. The original article has been updated.

## Figures and Tables

**Figure 1:**
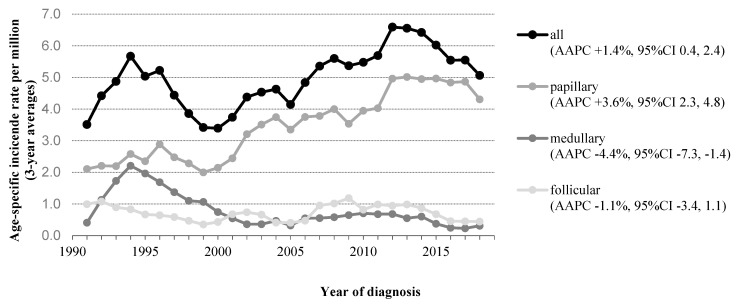
Time trends in incidence of patients aged 0–24 years with thyroid carcinoma in The Netherlands, 1990–2019. Abbreviations: AAPC, Average Annual Percent Change; CI, Confidence Interval. Three-year moving averages of the age-standardized incidence rate of thyroid carcinoma (standardized according to the World Standard Population) are shown. AAPC was estimated from a regression line, which was fitted to the natural logarithm of the rates using the year of diagnosis as a regressor variable.

**Figure 2:**
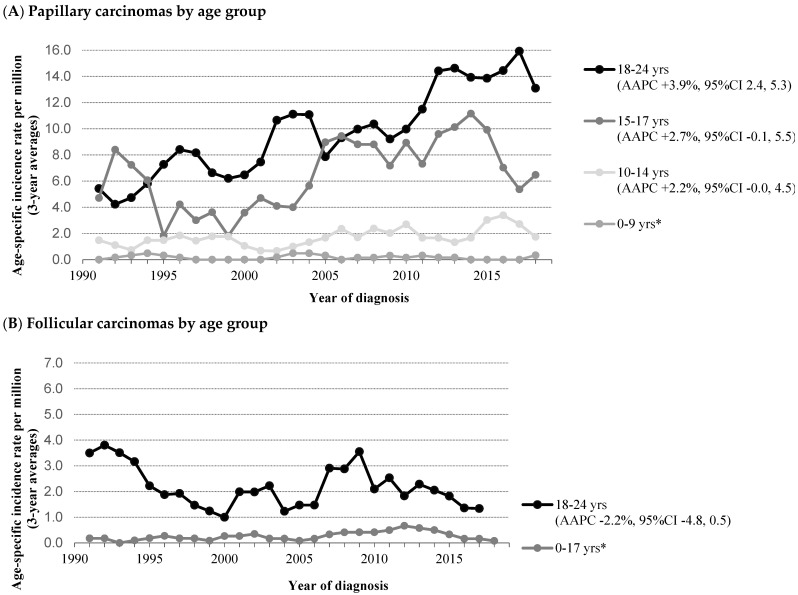

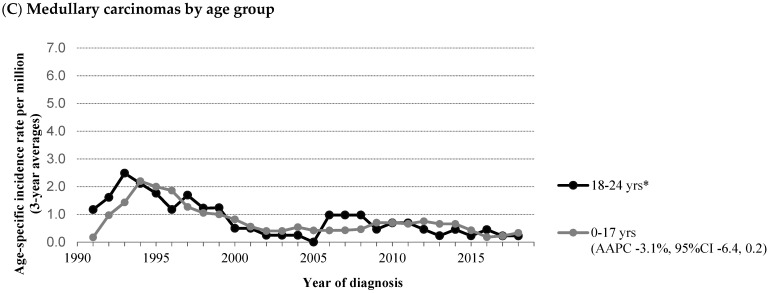
Time trends in incidence of patients aged 0–24 years with thyroid carcinoma by histology and age in The Netherlands, 1990–2019. (**A**) Papillary thyroid carcinoma. (**B**) Follicular thyroid carcinoma. (**C**) Medullary thyroid carcinoma. Abbreviations: AAPC, Average Annual Percent Change; CI, Confidence Interval. Three-year moving averages of the age-specific incidence rate of thyroid carcinoma are shown. The incidence rates of the patients 0–9 and 0–17 years are age-standardized according to the World Standard Population. AAPC was estimated from a regression line, which was fitted to the natural logarithm of the rates using year of diagnosis as a regressor variable. * Estimation of a reliable average annual percentage change was not possible because of *n* = 0 in >5 incidence years.
