# Supplementary material for: Opposite Incidence Trends for Differentiated and Medullary Thyroid Cancer in Young Dutch Patients over a 30-Year Time Span
Source: Cancers (Basel). 2021 Oct 12;13(20):5104. doi: 10.3390/cancers13205104 (PMC8534285; doi:10.3390/cancers13205104)
Supplement: Supplementary file 1 [file cancers-13-05104-s001.zip › cancers-1380328-supplementary.pdf]

Opposite Incidence Trends for Differentiated and Medullary Thyroid Cancer in Young Dutch Patients over a 30-Year Time Span

Chantal A. Lebbink, Medard F. M. van den Broek, Annemiek B. G. Kwast, Joep P. M. Derikx, Miranda P. Dierselhuis, Schelto Kruijff, Thera P. Links, A. S. Paul van Trotsenburg, Gerlof D. Valk, Menno R. Vriens, Annemarie A. Verrijn Stuart, Hanneke M. van Santen and Henrike E. Karim-Kos

Table S1. Absolute number of cases thyroid cancer according to age in young Dutch patients over a 30-year time span.

	PTC	FTC	MTC	Total
	N	*N*	*N*	*N*
**Total**	**594**	**128**	**114**	**839**
**Age**				
0			1	1
1			3	3
2			2	2
3			7	7
4	1		5	6
5	3		4	7
6	1		7	8
7		1	3	4
8	2	1	3	6
9	4		6	10
10	7		4	11
11	7	5	5	17
12	13	2	5	20
13	7		5	12
14	16	4	4	24
15	39	3	6	49
16	38	7	5	50
17	36	7	3	46
18	39	9	7	56
19	43	14	4	61
20	46	10	1	57
21	55	11	5	72
22	68	12	6	86
23	82	21	4	107
24	87	21	9	117

Abbreviations: FTC, follicular thyroid cancer; MTC, medullary thyroid carcinoma; PTC, papillary thyroid cancer.

Table S2. Incidence of thyroid carcinoma in children, adolescents and young adults aged 0–24 years in the Netherlands, 1990–2019.

Incidence of all thyroid carcinomas		Period of diagnosis 1990–1999	2000–2009	2010–2019	AAPC (%)	95% CI		*p*-value
	Average number of new cases/year	25	25	34				
	Incidence rate (per million)	4.4	4.6	5.8	**1.4**	0.4,	2.4	0.01
	Age (in years)							
0–9	Average number of new cases/year	3	2	1				
	Incidence rate (per million)	1.3	0.7	0.8	N.A.			
10–14	Average number of new cases/year	3	2	3				
	Incidence rate (per million)	3,4	2,3	3,0	1.0	–1.6	3.6	0.42
15–17	Average number of new cases/year	3	5	6				
	Incidence rate (per million)	6.1	8.4	10.1	2.7	–0.1	5.6	0.06
18–24	Average number of new cases/year	16	16	23				
	Incidence rate (per million)	10.6	11.9	15.7	**1.8**	0.7	3.0	0.003
Sex								
Boys	Average number of new cases / year	8	6	8				
	Incidence rate (per million)	2.7	2.0	3.0	0.9	–1.3,	3.1	0.41
Girls	Average number of new cases / year	18	19	25				
	Incidence rate (per million)	6.2	7.2	8.8	**1.8**	0.7,	2.9	0.002

Incidence of differentiated thyroid carcinomas		Period of diagnosis 1990–1999	2000–2009	2010–2019	AAPC (%)	95% CI		*p*-value
	Average number of new cases/year	19	22	31				
	Incidence rate (per million)	3.1	4.0	5.3	**2.6**	1.6	3.7	<0.001
	Age (in years)							
0–9	Average number of new cases/year	0.4	0.5	0.4				
	Incidence rate (per million)	0.2	0.2	0.2	NA			
10–14	Average number of new cases/year	2	2	3				
	Incidence rate (per million)	1.7	1.8	2.7	**2.9**	0.8	5.1	0.01
15–17	Average number of new cases/year	3	5	6				
	Incidence rate (per million)	4.8	7.8	9.5	**3.4**	0.4	6.5	0.03
18–24	Average number of new cases/year	14	15	23				
	Incidence rate (per million)	9.1	11.3	15.3	**2.5**	1.4	3.6	<0.001
Sex								
Boys	Average number of new cases/year	4	5	7				
	Incidence rate (per million)	1.4	1.7	2.4	**2.9**	1.0	4.8	0.01
Girls	Average number of new cases/year	14	17	24				
	Incidence rate (per million)	4.9	6.4	8.3	**2.7**	1.4	4.0	<0.001

Incidence of papillary thyroid carcinomas		Period of diagnosis 1990–1999	2000–2009	2010–2019	AAPC (%)	95% CI		*p*-value
	Average number of new cases/year	14	19	27				
	Incidence rate (per million)	2.4	3.3	4.6	**3.6**	2.3	4.8	<0.001
	Age (in years)							
0–9	Average number of new cases/year	0.3	0.4	0.4				
	Incidence rate (per million)	0.1	0.2	0.2	NA			
10–14	Average number of new cases/year	1	2	2				
	Incidence rate (per million)	1.5	1.5	2.1	2.2	–0.0	4.5	0.05
15–17	Average number of new cases/year	2	4	5				
	Incidence rate (per million)	4.3	6.7	8.1	2.7	–0.1	5.5	0.06
18–24	Average number of new cases/year	10	13	20				
	Incidence rate (per million)	6.4	9.3	13.4	**3.9**	2.4	5.3	<0.001

Sex								
Boys	Average number of new cases/year	3	4	6				
	Incidence rate (per million)	1.1	1.6	2.2	**3.4**	1.2	5.6	0.004
Girls	Average number of new cases / year	10	14	21				
	Incidence rate (per million)	3.6	5.2	7.1	**4.0**	2.0	5.9	<0.001

		Period of diagnosis			AAPC (%)			
**Incidence of follicular thyroid carcinomas**		**1990–1999**	**2000–2009**	**2010–2019**		**95% CI**		***p*-value**
	Average number of new cases/year	5	4	4				
	Incidence rate (per million)	0.8	0.7	0.7	–1.1	–3.4	1.1	0.31
	Age (in years)							
0–17	Average number of new cases/year	1	1	1				
	Incidence rate (per million)	0.2	0.3	0.3	NA			
18–24	Average number of new cases/year	4	3	3				
	Incidence rate (per million)	2.6	2.0	1.9	–2.2	–4.8	0.5	0.10
Sex								
Boys	Average number of new cases/year	1	1	1				
	Incidence rate (per million)	0.2	0.2	0.2	NA			
Girls	Average number of new cases/year	4	3	4				
	Incidence rate (per million)	1.3	1.2	1.2	–1.3	–4.0	1.4	0.32

		Period of diagnosis			AAPC (%)			
**Incidence of medullary thyroid carcinomas**		**1990–1999**	**2000–2009**	**2010–2019**		**95% CI**		***p*-value**
	Average number of new cases / year	7	3	2				
	Incidence rate (per million)	1.3	0.5	0.5	**–4.4**	–7.3	–1.5	0.003
	Age (in years)							
0–17	Average number of new cases / year	4	2	2				
	Incidence rate (per million)	1.2	0.5	0.5	–3.1	–6.4	0.2	0.06
18–24	Average number of new cases / year	2	1	1				
	Incidence rate (per million)	1.5	0.5	0.4	NA			
Sex								
Boys	Average number of new cases / year	4	1	1				
	Incidence rate (per million)	1.3	0.3	0.5	NA			
Girls	Average number of new cases / year	3	2	1				
	Incidence rate (per million)	1.3	0.7	0.5	–2.9	–6.1	0.4	0.08

Abbreviations: AAPC, average annual percentage change, NA, estimation of a reliable average annual percentage change was not possible because of $n = 0$ in >5 incidence years, 95% CI, 95% confidence interval.

Incidence rate is age-adjusted for the following groups: boys, girls, 0–9 years and the total group.

Statistical significant AAPCs are shown in bold.

Table S3.A. Characteristics of differentiated thyroid carcinoma patients aged 0–24 years in the Netherlands by age group, 1990–2019.

	Total		Age at diagnosis								
			<10 years		10–14 years		15–17 years		18–24 years		
	N	%	*N*	%	*N*	%	*N*	%	*N*	%	*P*-value
	722		13		61		130		518		
**Sex**											0.73
boys	162	22	3	23	17	28	30	23	112	22	
girls	560	78	10	77	44	72	100	77	406	78	
**Time period of diagnosis**											0.75
1990–99	186	26	4	31	16	26	27	21	139	27	
2000–09	223	31	5	38	18	30	46	35	154	30	
2010–18	313	43	4	31	27	44	57	44	225	43	
**Histology**											0.48
papillary carcinoma	594	82	11	85	50	82	113	87	420	81	
follicular carcinoma	128	18	2	15	11	18	17	13	98	19	
**T stage**[a]											0.05
1	198	28	5	45	14	24	33	27	146	29	
2	292	42	3	27	16	27	50	41	223	44	
3	145	21	2	18	20	34	26	21	97	19	
4	62	9	1	9	9	15	14	11	38	8	
unknown (3% of total)	25		2		2		7		14		
**N stage**[a]											0.01
0	379	56	7	54	25	41	56	47	291	60	
1	300	44	6	46	36	59	63	53	195	40	
unknown (6% of total)	43		0		0		11		32		
**Metastases**[a]											<0.001
no	606	97	10	91	45	83	117	98	434	98	
yes	20	3	1	9	9	17	3	3	7	2	
unknown (13% of total)	96		2		7		10		77		
**Thyroid carcinoma as second primary cancer**											0.02
yes	18	2	2	15	3	5	4	3	9	2	
no	704	98	11	85	58	95	126	97	509	98	

Abbreviations: *N*, number.

Characteristics of the study population with differentiated thyroid carcinoma, described as percentages, by different age groups: <10 years, 10–14 years, 15–17 years and 18–24 years. Differences among categorical variables were tested with the χ2 tests or the Monte Carlo estimate for the Exact test in case of small numbers.

[a] Tumor staging was recorded according to the TNM (Tumor, Node, Metastasis) classification system of the Union for International Cancer Control (UICC). The edition applicable at time of diagnosis of thyroid carcinoma was used.

Table S3. B. Characteristics of medullary thyroid carcinoma patients aged 0–24 years in the Netherlands by age group, 1990–2019.

	**Total**		**Age at diagnosis**				
			**<18 years**		**18–24 years**		
	N	%	*N*	%	*N*	%	*p*-value
	114		78		36		
**Sex**							0.51
boys	55	48	36	46	19	53	
girls	59	52	42	54	17	47	
**Time period of diagnosis**							0.67
1990–99	66	58	43	55	23	64	
2000–09	25	22	18	23	7	19	
2010–18	23	20	17	22	6	17	
**T stage**[a]							0.01
1	83	78	63	85	20	61	
2	12	11	7	9	5	15	
3	6	6	3	4	3	9	
4	6	6	1	1	5	15	
unknown (6% of total)	7		4		3		
**N stage**[a]							0.001
0	67	72	54	83	13	46	
1	26	28	11	17	15	54	
unknown (18% of total)	21		13		8		
**Metastases**[a]							0.14
no	75	94	55	96	20	87	
yes	5	6	2	4	3	13	
unknown (30% of total)	34		21		13		

Abbreviations: *N*, number.

Characteristics of the study population with medullary thyroid carcinoma, described as percentages, by different age groups: <18 years and 18–24 years. Differences among categorical variables were tested with the $\chi 2$ tests or the Monte Carlo estimate for the Exact test in case of small numbers.

[a] Tumor staging was recorded according to the TNM (Tumor, Node, Metastasis) classification system of the Union for International Cancer Control (UICC). The edition applicable at time of diagnosis of thyroid carcinoma was used.

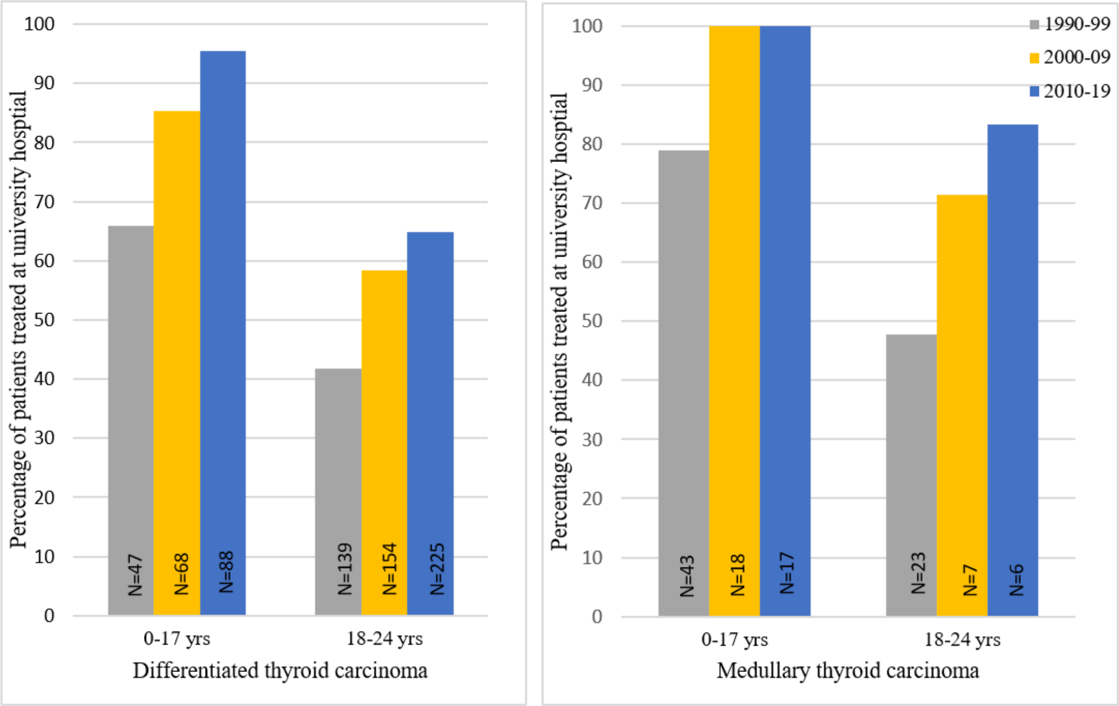

Figure S1. Proportion of patients with thyroid carcinoma aged <18 years and aged 18–24 years, treated at a university center.

Abbreviations: *N*, number.

Percentage of patients treated at a university hospital, by age group and time period. Patients were classified as treated in an university hospital if they received thyroidectomy and/or radioiodine treatment in an university hospital. One patient with PTC has been excluded from this analysis, because of treatment abroad.
